# Phloretin Attenuates Allergic Airway Inflammation and Oxidative Stress in Asthmatic Mice

**DOI:** 10.3389/fimmu.2017.00134

**Published:** 2017-02-13

**Authors:** Wen-Chung Huang, Li-Wen Fang, Chian-Jiun Liou

**Affiliations:** ^1^Graduate Institute of Health Industry Technology, Research Center for Industry of Human Ecology, Research Center for Chinese Herbal Medicine, College of Human Ecology, Chang Gung University of Science and Technology, Taoyuan, Taiwan; ^2^Division of Allergy, Asthma, and Rheumatology, Department of Pediatrics, Chang Gung Memorial Hospital, Taoyuan, Taiwan; ^3^Department of Nutrition, I-Shou University, Kaohsiung, Taiwan; ^4^Department of Nursing, Research Center for Chinese Herbal Medicine, Chang Gung University of Science and Technology, Taoyuan, Taiwan

**Keywords:** asthma, cytokine, eosinophil, oxidative stress, phloretin

## Abstract

Phloretin (PT), isolated from the apple tree, was previously demonstrated to have antioxidative and anti-inflammatory effects in macrophages and anti-adiposity effects in adipocytes. Inflammatory immune cells generate high levels of reactive oxygen species (ROS) for stimulated severe airway hyperresponsiveness (AHR) and airway inflammation. In this study, we investigated whether PT could reduce oxidative stress, airway inflammation, and eosinophil infiltration in asthmatic mice, and ameliorate oxidative and inflammatory responses in tracheal epithelial cells. BALB/c mice were sensitized with ovalbumin (OVA) to induce asthma symptoms. Mice were randomly assigned to the five experimental groups: normal controls; OVA-induced asthmatic mice; and OVA-induced mice injected intraperitoneally with one of the three PT doses (5, 10, or 20 mg/kg). In addition, we treated inflammatory human tracheal epithelial cells (BEAS-2B cells) with PT to assess oxidative responses and the levels of proinflammatory cytokines and chemokines. We found that PT significantly reduced goblet cell hyperplasia and eosinophil infiltration, which decreased AHR, inflammation, and oxidative responses in the lungs of OVA-sensitized mice. PT also decreased malondialdehyde levels in the lung and reduced Th2 cytokine production in bronchoalveolar lavage fluids. Furthermore, PT reduced ROS, proinflammatory cytokines, and eotaxin production in BEAS-2B cells. PT also suppressed monocyte cell adherence to inflammatory BEAS-2B cells. These findings suggested that PT alleviated pathological changes, inflammation, and oxidative stress by inhibiting Th2 cytokine production in asthmatic mice. PT showed therapeutic potential for ameliorating asthma symptoms in the future.

## Introduction

Allergic asthma is a complex, chronic inflammatory airway disease. Patients with sudden, acute asthma attacks exhibit shortness of breath, dry coughing, chest tightness, paroxysmal wheezing due to airway obstruction, troubled breathing, and even death (1). In recent years, public health surveys found that the mortality due to acute asthma was increasing in developing countries (2). Asthma progression involves pathology in the respiratory system, characterized by airway smooth muscle proliferation, the airway narrowing, and goblet cell hyperplasia, accompanied by mucus hypersecretion, pulmonary eosinophilia, and airway hyperresponsiveness (AHR) (3, 4).

Recent studies have shown that the bronchial asthma is an inappropriate anaphylactic immune response to chronic lung inflammatory disease. Several immune cells, including Th2 cells, mast cells, eosinophils, and airway epithelial cells, secrete cytokines, chemokines, and inflammatory mediators that damage lung tissue and lead to the restricted breathing (4). Importantly, activated Th2 cells release additional cytokines, including IL-4, IL-5, and IL-13, to stimulate IgE production, which then causes mast cell activation, goblet cell hyperplasia, with excessive mucus secretion, eosinophil infiltration, and AHR (5). Furthermore, inflammatory immune cells, including eosinophils, neutrophils, monocytes, and epithelial cells, generate high levels of reactive oxygen species (ROS) to damage lung tissue (6, 7). Hence, asthma progression may be ameliorated by regulating the activity of Th2 cells and reducing the infiltration of inflammatory immune cells.

Phloretin (PT), a flavonoid of the chalcone class, is found in the fruit, leaves, and bark of apple trees (8). PT has many biological functions; it was shown to regulate glucose transporters, promote apoptosis in tumor cells, and enhance lipid metabolism to defend against obesity (9, 10). PT was also found to have antioxidase activity, which reduced oxidative damage in a rat model of cerebral ischemia (11).

Our earlier studies demonstrated that PT reduced the inflammatory and oxidative stress in LPS-induced acute lung injury mice (12). PT also suppressed inflammatory mediator expression in IL-1β-stimulated lung epithelial cells (10). Therefore, we reasoned that PT might improve asthma by blocking inflammatory responses and oxidative stress. In the current study, we treated asthmatic mice with intraperitoneal injections of PT to investigate whether PT could ameliorate the pathogenesis of asthma. We evaluated inflammation, oxidative stress, and immune function in asthmatic mice.

## Animals and Methods

### Animals

Female BALB/c mice (6–8 weeks old) were obtained from the National Laboratory Animal Center in Taiwan. All mice were housed in air-conventional animal housing with food and water *ad libitum*. Animal care and experimental procedures were performed in accordance with the guidelines of the Laboratory Animal Care Committee of Chang Gung University of Science and Technology (IACUC approval number: 2014-023).

### Sensitization, Airway Challenge, and Drug Treatment

Phloretin (extracted from apple wood; ≥99% purity by HPLC; purchased from Sigma-Aldrich, St. Louis, MO, USA) was dissolved in DMSO. Mice were sensitized with intraperitoneal injections (200 µl) that contained 50 µg ovalbumin (OVA; Sigma, St. Louis, MO, USA) mixed with 2 mg aluminum hydroxide (Thermo, Rockford, IL, USA) in normal saline. Injections were performed on days 1–3 and 14, as described previously (13). On days 14, 17, 20, 23, and 27, mice were challenged with inhalations of 2% OVA for 30 min, delivered with an ultrasonic nebulizer (DeVilbiss Pulmo-Aide 5650D, USA). One hour before the OVA inhalation challenge, mice were treated with or without intraperitoneal injections of PT. Then, the OVA challenge was followed by an AHR assay. Mice were randomly divided into five groups (12 mice each), as follows: healthy mice sensitized with normal saline and were given equal volume of DMSO by intraperitoneal injection (N group) (2); mice sensitized with OVA and were given equal volume of DMSO by intraperitoneal injection (OVA group); OVA-sensitized mice treated with 5, 10, or 20 mg/kg PT (PT5, PT10, and PT20 groups, respectively).

### Measurement of AHR

Airway hyperresponsiveness was measured to assess airway function after inhaling aerosolized methacholine, as described previously (14). All mice inhaled 0–40 mg/ml methacholine for 3 min; then, mice were placed in a single-chamber, whole-body plethysmograph (Buxco Electronics, Troy, NY, USA) to record the enhanced pause (Penh), a variable for estimating the AHR.

### Splenocyte Cultures and Serum Collection

Splenocytes (5 × 10^6^ cells/ml) were isolated and cultured in RPMI 1640 medium containing 10% FBS (Biological Industries, Haemek, Israel), 100 U/ml penicillin and streptomycin, and 100 µg/ml OVA for five continuous days. The supernatants were collected, and cytokine concentrations were measured, as previously described (13).

Blood collected from the orbital vascular plexus and centrifuged at 6,000 rpm for 5 min. The serum collected and stored at −80°C as previously described. Serum was measure OVA-specific antibodies with an enzyme-linked immunosorbent assay (ELISA).

### Histological Analysis of Lung Tissue

Lung tissues were fixed in 10% formalin, embedded in paraffin, and cut into sections 6 μm thick. Sections were stained with hematoxylin and eosin (HE), to examine eosinophil infiltration Inflammatory index calculated and evaluated score using fivepoint scoring system as described previously (15). The score were considered by the inflammatory cell infiltration on perivascular regions and peribronchial of lungs. Furthermore, tracheal sections were stained with the periodic acid-Schiff (PAS) staining system (Sigma), to measure goblet cell hyperplasia, as described previously (14).

### Malondialdehyde (MDA) Activity

We measured MDA activity in lung tissues with the lipid peroxidation assay kit, according to the manufacturer’s instructions (Sigma). MDA activity was measured as nanomolars of MDA in milligram weight of wet tissue with a Multi-Mode Microplate Reader (BioTek SynergyHT, Bedfordshire, UK).

### Glutathione (GSH) Assay

To detect glutathione levels in lung tissues, we employed a glutathione assay kit, according to the manufacturer’s instructions (Sigma). The kit measured total glutathione, glutathione disulfide, and reduced glutathione. The glutathione reaction was measured spectrophotometrically at 412 nm with a microplate reader (Multiskan FC, Thermo, Waltham, MA, USA). Glutathione expressed as nanomolars of MDA in milligram weight of wet tissue.

### Bronchoalveolar Lavage Fluid (BALF) and Cell Counting

Mice were anesthetized and sacrificed to collect BALF, as described previously (16). Mouse tracheas were intubated with an indwelling needle to wash the lungs with normal saline. The supernatants would assay cytokine and chemokine levels. We used Liu stain solution (Polysciences, Inc., Taipei, Taiwan) to differentiate cell morphology and determine cells counts.

### Enzyme-Linked Immunosorbent Assay

Serum OVA-specific antibodies, including IgE and IgG1, were measured with a specific ELISA kit (BD Biosciences). Serum of OVA-sensitized mice makes OVA-IgG1 standard curves for determining the concentrations of IgG1. Serum was diluted fivefold to detect the absorbance of OVA-IgE at an optical density of 450 nm, as previously described (14). Furthermore, the cell culture supernatants and BALF were used to measure with specific ELISA kits to detect the concentrations of CCL5, CCL11, CCL24, CCL26, intercellular adhesion molecule 1 (ICAM-1), IL-4, IL-5, IL-6, IL-8, IL-13, MCP-1, and tumor necrosis factor-α (TNF-α), according to the manufacturer’s instructions (R&D Systems, Minneapolis, MN, USA), as previously described (16).

### RNA Isolation and Real-time PCR

Lung tissues were homogenized, and RNA was extracted with TRIzol reagent (Life Technologies, Carlsbad, CA, USA); complementary DNA (cDNA) was generated from 1 µg total RNA using cDNA synthesis kit (Life Technologies). cDNA gene expression assayed by real-time PCR performed with the SYBR Green system (Fermentas, Thermo, Waltham, MA, USA) and specific primers (Table 1) for the genes by the spectrofluorometric thermal cycler (iCycler; Bio-Rad Laboratories, Hercules, CA, USA). The average of gene cycle threshold (*C*_t_) was measured for each experiment. Relative cDNA expressions $\left(2^{-\Delta \Delta C_{\text{t}}}\right)$ for the specific genes were determined by the compared *C*_t_ method, which generates ΔΔ*C*_t_ as the discrepancy between the housekeeping genes β-actin and the specific gene for each sample.

**Table 1:** **Primers used in real-time PCR analyses of cytokine and chemokine mRNA expression levels**.

Gene	Primer forward	Primer reverse
CCL11	GGCTTCATGTAGTTCCAGAT	CCATTGTGTTCCTCAATAATCC
CCL24	AGGCAGTGAGAACCAAGT	GCGTCAATACCTATGTCCAA
COX-2	ACCAGCAGTTCCAGTATCAGA	CAGGAGGATGGAGTTGTTGTAG
Gob5	AATGGATGAATGGCTCAGTGAT	TATTGTAGGAGGATGCGTTGTC
Intercellular adhesion molecule 1	AACAGAATGGTAGACAGCAT	TCCACCGAGTCCTCTTAG
IFN-γ	CAGCAACAACATAAGCGTCATT	ACCTCAAACTTGGCAATACTCA
IL-4	TCCGTGCTTGAAGAAGAACTC	GTGATGTGGACTTGGACTCATT
IL-5	ATCCTCCTGCCTCCTCTTCC	GGTTCCATCTCCAGCACTTCA
IL-13	GCTCCAGCATTGAAGCAGTG	CGTGGCAGACAGGAGTGTT
iNOS	TTCCACAACCACCTCAAGCA	TTAAGGCATCACAGTCCGAGTC
MUC5AC	AATGCTGGTGCCTGTGTCT	CCTCCTATGCCATCTGTTGTG
β-Actin	AAGACCTCTATGCCAACACAGT	AGCCAGAGCAGTAATCTCCTTC

### BEAS-2B Cell Culture and PT Treatment

Phloretin was dissolved in DMSO at a concentration of 100 mM to produce a stock solution. After dilution, the DMSO component was ≤0.1% of the experimental culture medium. We seeded immortalized human bronchial epithelial cells (BEAS-2B) into 24-well plates with DMEM/F12 medium. Cell cultures were pretreated with PT (3–30 µM) for 1 h, then treated with 10 ng/ml TNF-α for 24 h, or 10 ng/ml TNF-α and 20 ng/ml IL-4 for 24 h. The supernatants were collected, and the levels of cytokines or chemokines were determined with specific ELISA kits.

### Cell–Cell Adhesion Assay

BEAS-2B cells were treated with PT and stimulated with TNF-α for 24 h. The human monocytic cell line, THP-1, was cultured and stained with calcein-AM solution (Sigma) for 0.5 h, then cocultured with BEAS-2B cells for 1 h. Adherent cells were observed and evaluated with fluorescence microscopy (Olympus, Tokyo, Japan).

### Determination of ROS Production

TNF-α-stimulated BEAS-2B cells treated with PT were seeded in 96-well plates for 24 h. Next, cells were stained with 20 µM 2′,7′-dichlorofluorescin diacetate (DCFH-DA) for 30 min. Then, cells were lysed and analyzed with a Multi-Mode Microplate Reader (BioTek synergy HT); fluorescence was evaluated by exciting at 485 nm and measuring emission at 528 nm. Furthermore, intracellular ROS was visualized with a fluorescence microscope (Olympus).

### Western Immunoblot Analysis

Lung tissue proteins were quantified and separated on 10% SDS polyacrylamide gels. The proteins were transferred to polyvinylidene fluoride (PVDF) membranes (Millipore, Billerica, MA, USA) and incubated with primary antibodies overnight at 4°C. Then, membranes were washed and incubated with secondary antibodies. Finally, PVDF membranes were treated with Luminol/Enhancer Solution (Millipore) to detect antibody signals with the BioSpectrum 600 system (UVP, Upland, CA, USA). Primary antibodies included anti-HO-1, anti-Nrf2, and anti-Lamin B1 (Santa Cruz, CA, USA); β-actin expression was evaluated as a loading control (Sigma).

### Statistical Analysis

Data were assessed with one-way analysis of variance, followed by the Tukey–Kramer *post hoc* test for multiple comparisons. All values represent the mean ± SEM. Values of *p* < 0.05 were considered significant.

## Results

### Effect of PT on Allergen-Induced AHR in Mice

**Figure 1:**
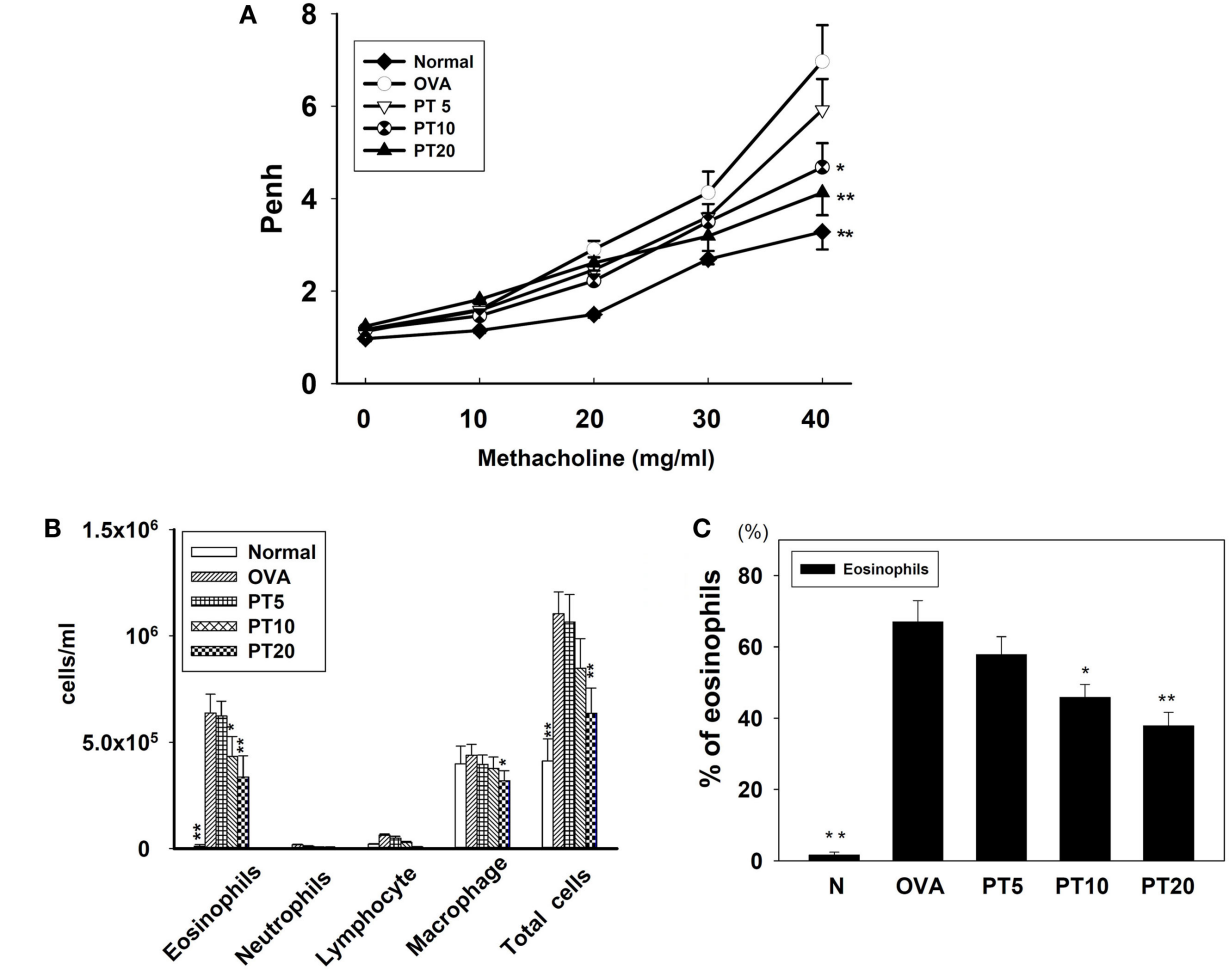
**The effect of phloretin (PT) on airway hyperresponsiveness (AHR) and cell counts in bronchoalveolar lavage fluid (BALF) of asthmatic mice**. **(A)** Changes in AHR (Penh values) with inhalation of increasing methacholine doses (10–40 mg/ml) in normal (N) and OVA-stimulated (OVA) mice, without or with PT (PT5-20) treatment (*n* = 12 mice/group, measured in three independent experiments). **(B)** Numbers of inflammatory cells and total cells in BALF with increasing PT doses; **(C)** percentage of eosinophils in BALFs from OVA-sensitive mice, treated or untreated with PT. All data are presented as means ± SEM. *p < 0.05 compared to OVA control group. **p < 0.01 compared to the OVA control group. Three independent experiments were analyzed and compared with the OVA-sensitive mice.

We evaluated whether PT could improve shortness of breath and abnormal airflow in the airways of asthmatic mice. Airway function was evaluated as the AHR, and the Penh value was calculated to determine the AHR. Mice were placed into the single-chamber whole-body plethysmograph to record the Penh, during inhalation of various methacholine doses (0–40 mg/ml). We found that, upon inhaling methacholine, the Penh value increased in a dose-dependent manner. The Penh values were greater in OVA-sensitized mice compared to normal mice (Figure 1A). At 40 mg/ml of inhaled methacholine, PT-treated asthmatic mice showed significantly lower Penh values (PT5, 5.9 ± 0.66, *p* = 0.32; PT10, 4.41 ± 0.58, *p* < 0.05; PT20, 3.94 ± 0.48, *p* < 0.01) compared to asthmatic mice in the OVA-sensitized group (OVA, 6.96 ± 0.78). Hence, PT could significantly diminish AHR in asthmatic mice.

### PT Effect on Inflammatory Cells in BALF

We counted various types of inflammatory cells to assess whether PT decreased the inflammatory response in asthmatic mice by reducing the numbers of inflammatory cells in BALF (Figure 1B). Asthmatic mice treated with PT had significantly reduced numbers of eosinophils and total cells compared to the OVA group (eosinophils: PT5: 6.2 × 10^5^ ± 6.9 × 10^4^, *p* = 0.57; PT10: 4.3 × 10^5^ ± 9.3 × 10^3^, *p* < 0.05; PT20: 3.3 × 10^5^ ± 1.1 × 10^4^, *p* < 0.01 vs. OVA: 6.4 × 10^5^ ± 8.9 × 10^4^) (total cells: PT5: 1.0 × 10^6^ ± 1.3 × 10^5^, *p* = 0.84; PT10: 8.4 × 10^5^ ± 1.3 × 10^4^, *p* = 0.24; PT20: 6.3 × 10^5^ ± 1.2 × 10^4^, *p* < 0.01 vs. OVA: 1.1 × 10^6^ ± 1.0 × 10^5^). Furthermore, the proportion of eosinophils in BALF derived from the P10 or the P20 group was significantly reduced compared to the proportion in the OVA group (Figure 1C).

### PT Modulated Chemokine and Cytokine Levels in BALF and Lung Tissue

**Figure 2:**
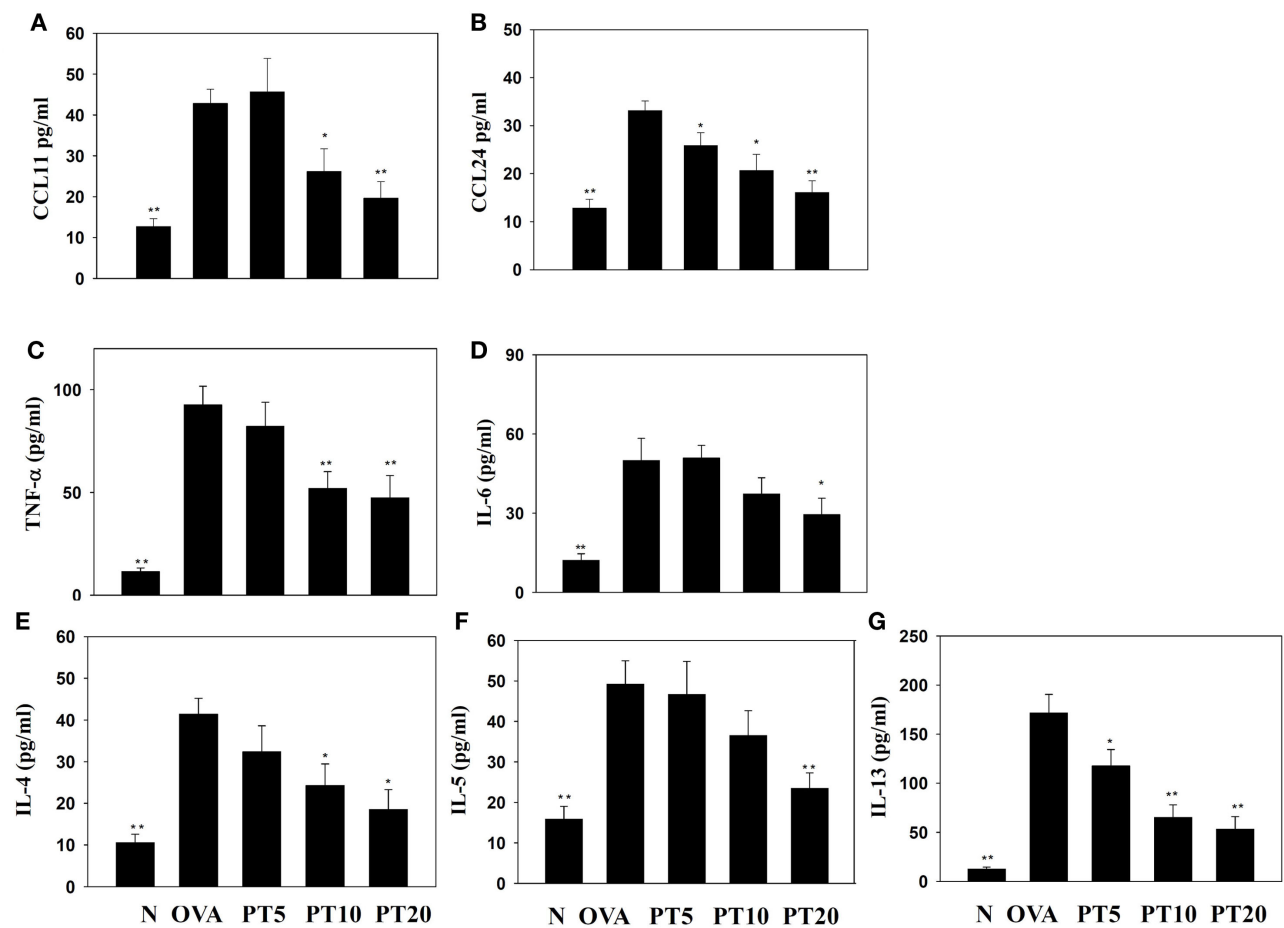
**Effects of phloretin (PT) on the levels of cytokines and chemokines in bronchoalveolar lavage fluid (BALF)**. The concentrations of **(A)** CCL11, **(B)** CCL24, **(C)** tumor necrosis factor-α (TNF-α), **(D)** IL-6, **(E)** IL-4, **(F)** IL-5, and **(G)** IL-13 were measured by enzyme-linked immunosorbent assay in BALF from normal (N) and OVA-stimulated (OVA) mice, without or with PT (PT5-20) treatment. All data are presented as the means ± SEM. *p < 0.05 compared to the OVA control group. **p < 0.01 compared to the OVA control group. Three independent experiments were analyzed and compared with the OVA-sensitive mice.

**Figure 3:**
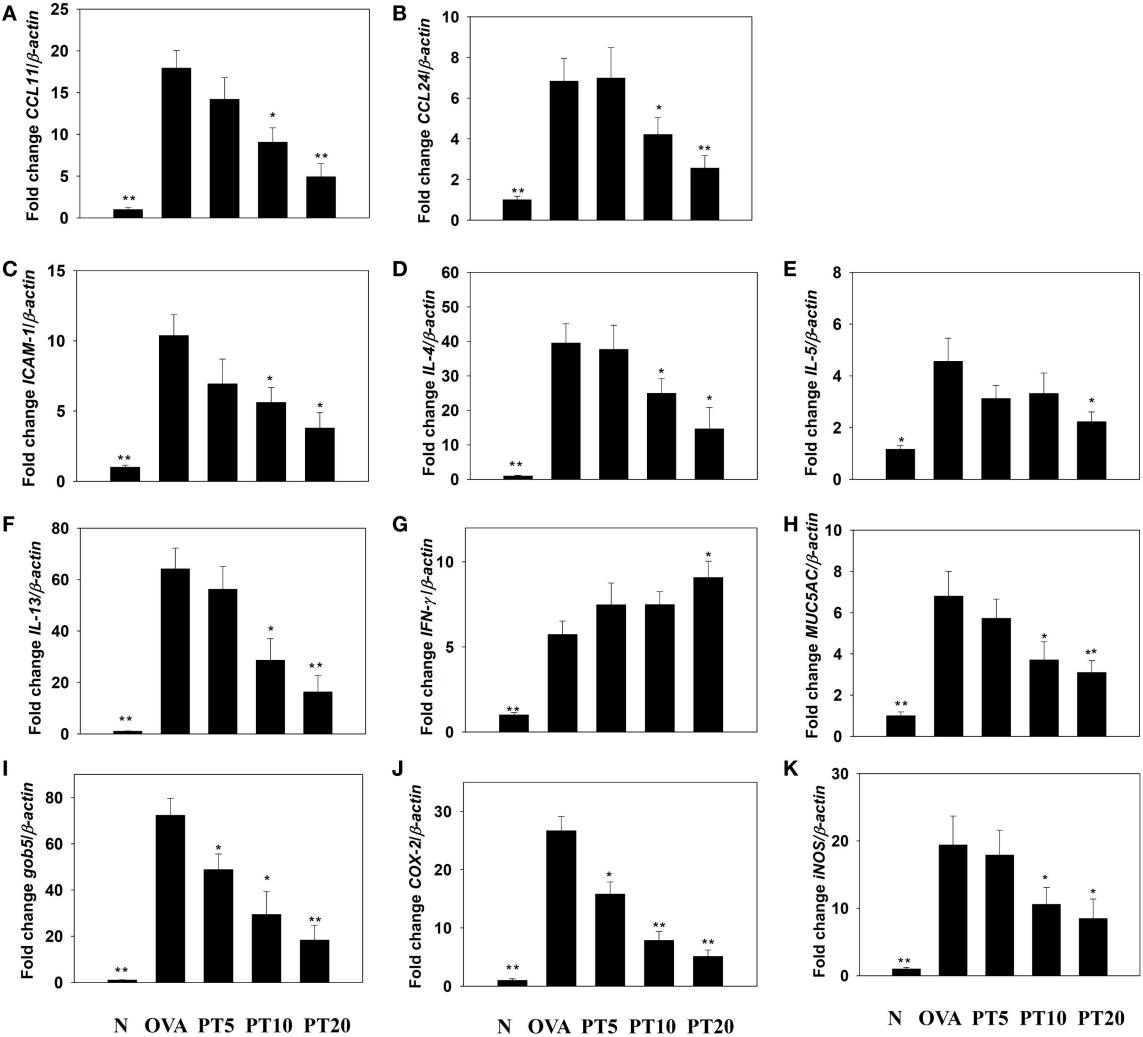
**Phloretin (PT) effects on cytokine, chemokine, and inflammatory mediator mRNA expression in the lungs**. Gene expression levels of **(A)** CCL11, **(B)** CCL24, **(C)** ICAM-1, **(D)** IL-4, **(E)** IL-5, **(F)** IL-13, **(G)** IFN-γ, **(H)** MUC5AC, **(I)** Gob5, **(J)** COX-2, and **(K)** iNOS were determined by real-time RT-PCR of RNA extracted from lung tissues of normal (N) and OVA-stimulated (OVA) mice, without or with PT (PT5-20) treatment. Fold changes in expression were measured relative to the β-actin expression (internal control). Data are presented as the mean ± SEM. *p < 0.05 compared to OVA control mice. **p < 0.01 compared to OVA control mice. Three independent experiments were analyzed and compared with the OVA-sensitive mice.

The levels of cytokines and chemokines in BALF were determined by ELISA (Figure 2). Our results showed that PT could significantly suppress IL-4 levels compared to OVA-sensitization alone (PT5: 32.4 ± 6.2 pg/ml, *p* = 0.33; PT10: 24.3 ± 5.2 pg/ml, *p* < 0.05; PT20: 18.5 ± 4.8 pg/ml, *p* < 0.05 vs. OVA: 41.3 ± 3.8 pg/ml). In addition, the PT groups had significantly decreased the levels of CCL11, CCL24, TNF-α, IL-6, IL-5, and IL-13 compared to the OVA group. A real-time PCR analysis of the expression of genes in lung tissue showed that PT could significantly decrease the levels of CCL11, CCL24, and ICAM-1 expression compared to the levels in OVA-sensitized asthmatic mice. PT also inhibited IL-4, IL-5, IL-13, MUC5AC, Gob5, iNOS, and COX-2 gene expression. Furthermore, PT increased the expression of IFN-γ compared to the expression in asthmatic mice (Figure 3).

### Effect of PT on Eosinophil Infiltration and Goblet Cell Hyperplasia in Lungs

**Figure 4:**
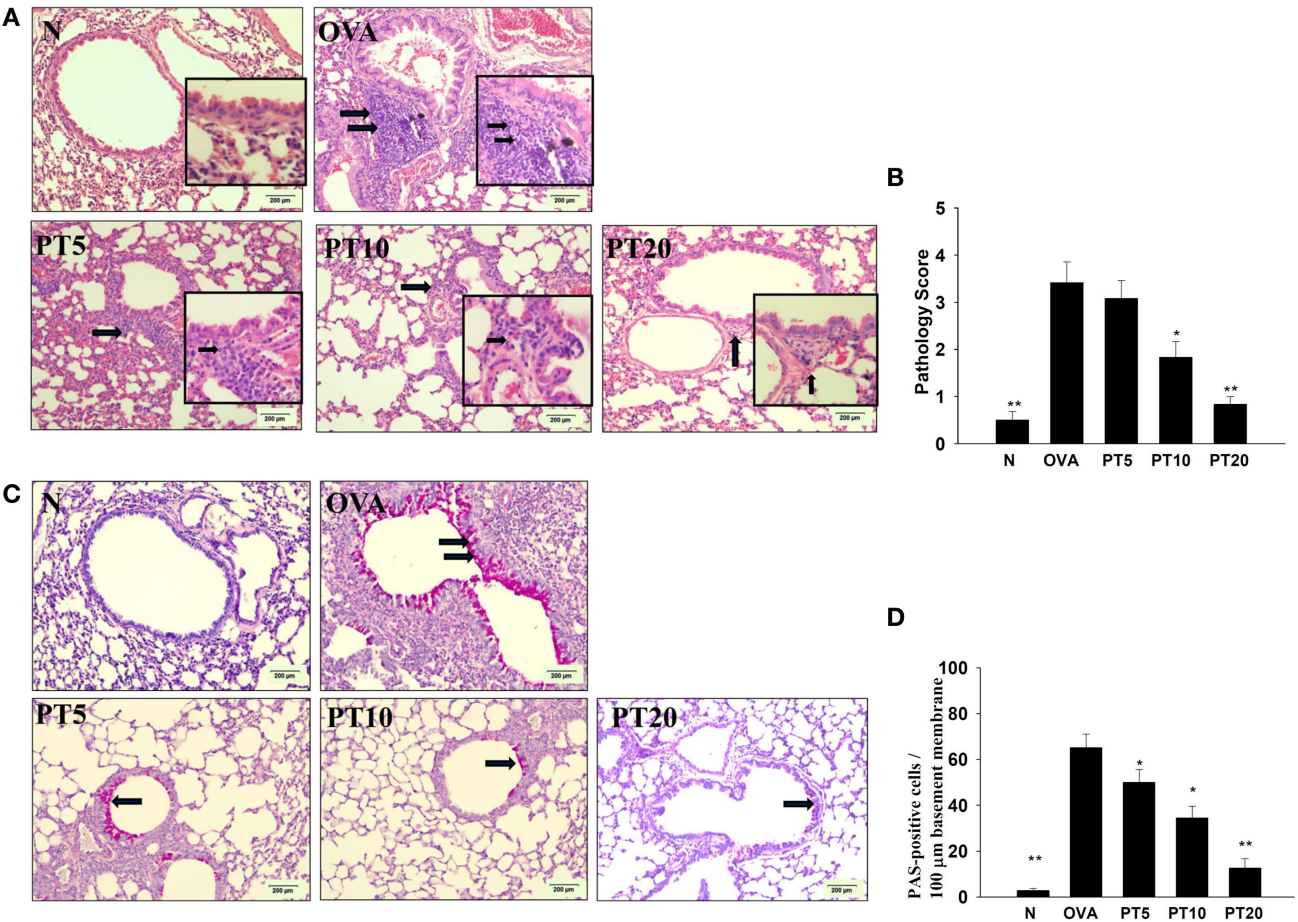
**Phloretin (PT) effects on asthmatic lung tissue**. Histological sections of lung tissues from normal (N) and OVA-stimulated (OVA) mice, without or with PT (PT5-20) treatment. **(A)** PT reduced eosinophil infiltration; eosinophils are indicated with arrows (hematoxylin and eosin stain; 200× magnification). Amplification sections (400× magnification) were shown for the indicated areas. **(B)** Scoring of inflammation *via* pathological evaluation of inflammatory cell infiltration in lung sections. **(C)** Periodic acid-Schiff (PAS)-stained lung sections show goblet cell hyperplasia; goblet cells are indicated with arrows (200× magnification). **(D)** Results were expressed as the number of PAS-positive cells per 100 µm of basement membrane. All data are presented as the means ± SEM. *p < 0.05 compared to the OVA control group. **p < 0.01 compared to the OVA control group. Three independent experiments were analyzed and compared with the OVA-sensitive mice.

Eosinophil infiltration in the lungs was evaluated with HE staining. Compared to normal mice, OVA-sensitized mice exhibited more infiltrating eosinophils between the bronchus and blood vessels (Figures 4A,B). PT reduced eosinophil infiltration in the lungs of asthmatic mice. We evaluated tracheal goblet cell hyperplasia with PAS staining. We found that PT could inhibit goblet cell hyperplasia compared to untreated, OVA-sensitized asthmatic mice (Figures 4C,D).

### Effects of PT on GSH and MDA Activity in the Lung

**Figure 5:**
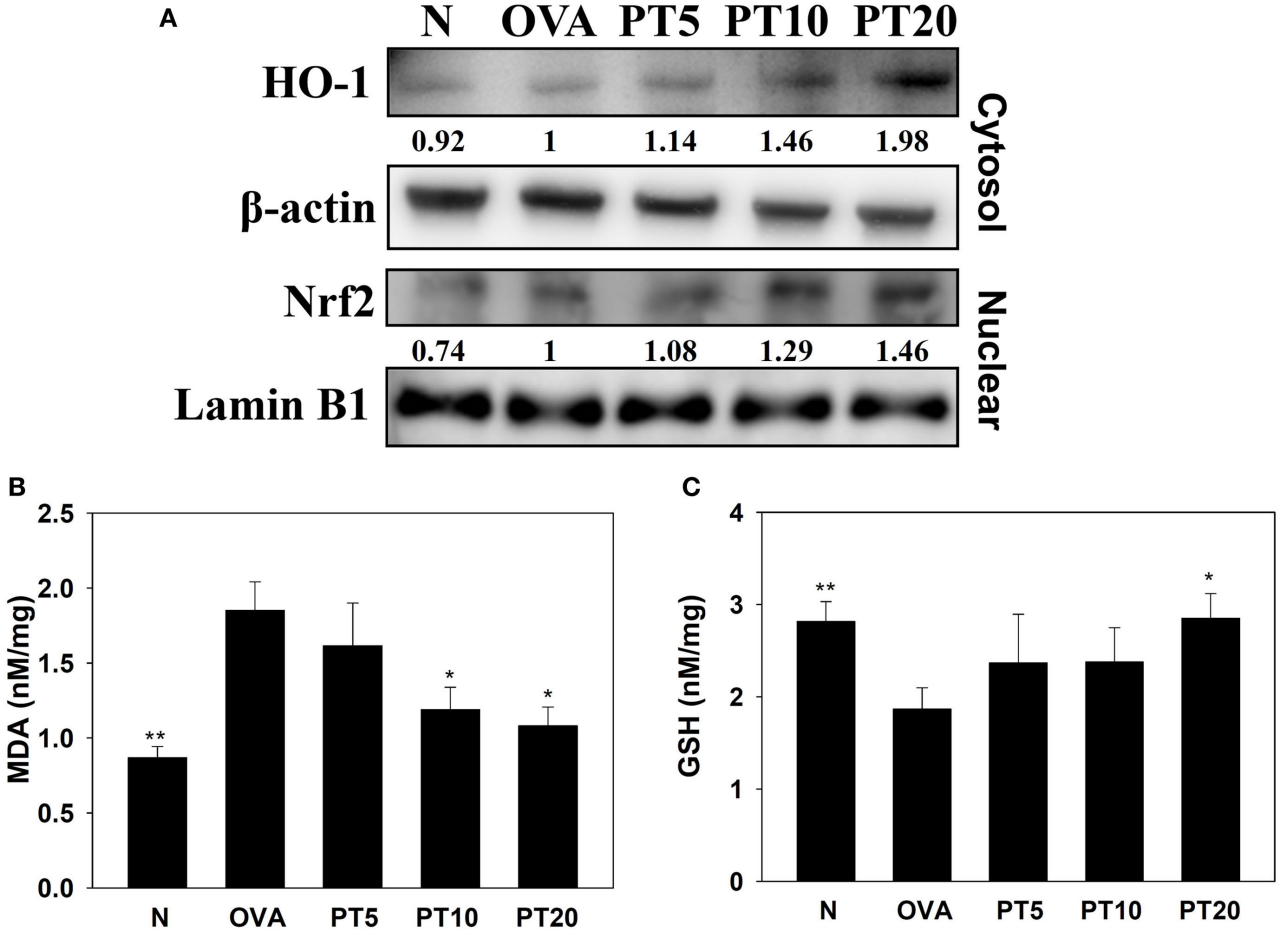
**Phloretin (PT) effects on oxidative stress factors**. **(A)** Western blot shows PT modulation of HO-1 and Nrf2 expression in lung tissue of normal (N) and OVA-stimulated (OVA) mice, without or with PT (PT5-20) treatment. **(B)** Malondialdehyde (MDA) activity and **(C)** GSH activity in lung tissues of mice. Data are presented as the mean ± SEM. *p < 0.05 compared to OVA control mice. **p < 0.01 compared to OVA control mice. Three independent experiments were analyzed and compared with the OVA-sensitive mice.

Acute asthma attacks can also cause oxidative stress. Previous studies showed that the expression of antioxidant HO-1 could protect and decrease lung damage during oxidative stress (17). We found that the lungs in PT-treated mice had increased HO-1 expression of lung compared to asthmatic mice. Nrf2, is a transcription factor, could translocate into the nucleus to promote HO-1 expression for antioxidant response. PT could increase nuclear Nrf2 expression of lung cells compared to OVA-sensitized asthmatic mice (Figure 5A). We also found that the OVA-sensitized asthmatic mice had significantly increased MDA activity and decreased GSH levels in lung tissues compared to the levels in normal mice (Figures 5B,C). However, PT significantly reduced MDA activity and promoted GSH production in lung tissues, compared to the levels in OVA-sensitized asthmatic mice.

### PT Modulated Splenocyte Cytokine Levels and Serum OVA-Specific Antibody

**Figure 6:**
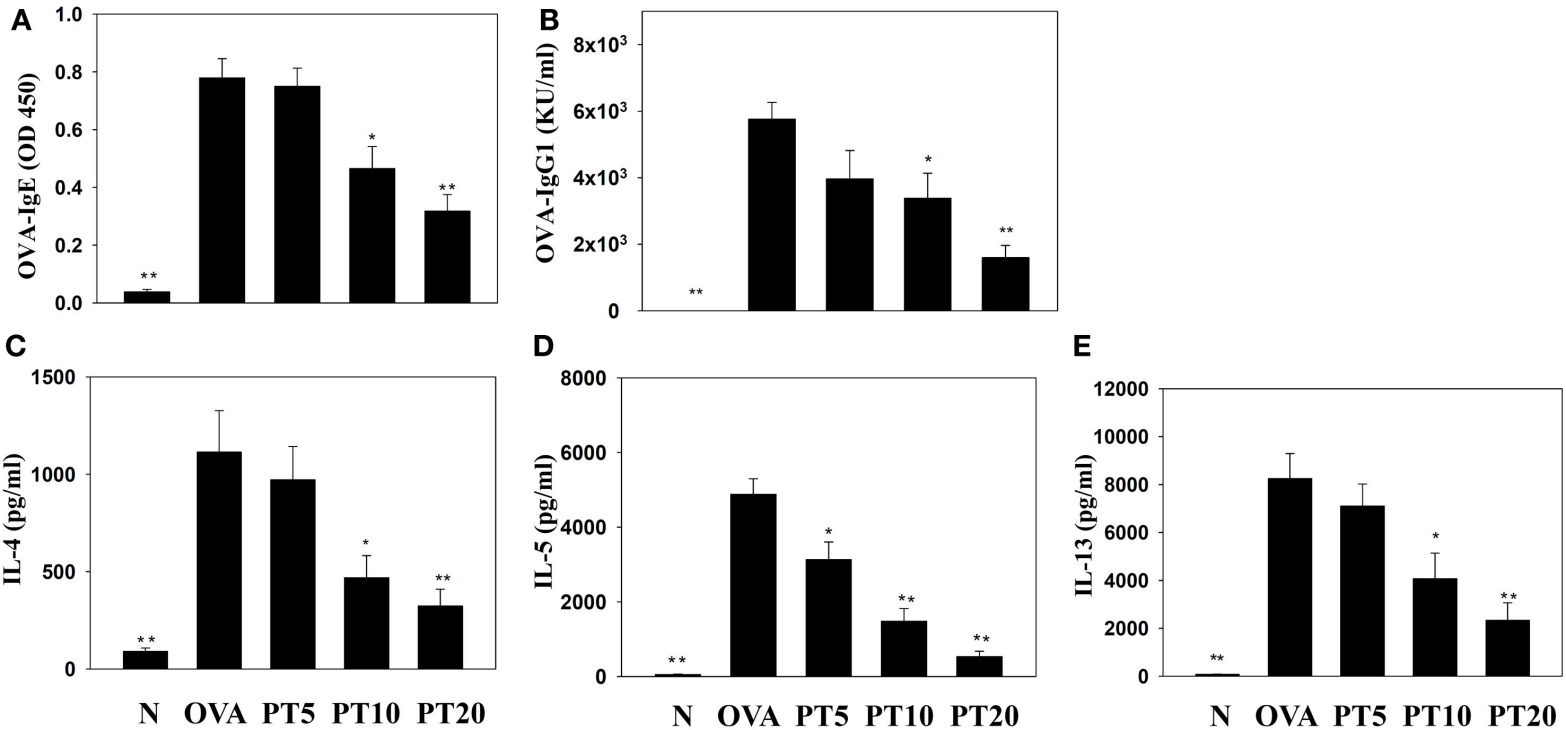
**Phloretin (PT) effects on OVA-specific antibodies in serum**. Serum levels of **(A)** OVA-IgE and **(B)** OVA-IgG1 are shown from normal (N) and OVA-stimulated (OVA) mice, without or with PT (PT5-20) treatment. PT also changed the cytokine levels produced by OVA-activated splenocytes, including **(C)** IL-4, **(D)** IL-5, and **(E)** IL-13. All data are presented as the means ± SEM. *p < 0.05 compared to the OVA control group. **p < 0.01 compared to the OVA control group. Three independent experiments were analyzed and compared with the OVA-sensitive mice.

Splenocyte culture supernatant analyses showed that PT significantly attenuated the levels of IL-4, IL-5, and IL-13, compared to untreated OVA-sensitized cells. PT also significantly decreased the levels of OVA-IgE and OVA-IgG1 in the serum of OVA-sensitized asthmatic mice (Figure 6).

### PT Suppressed Inflammatory Mediators in Activated BEAS-2B Cells

**Figure 7:**
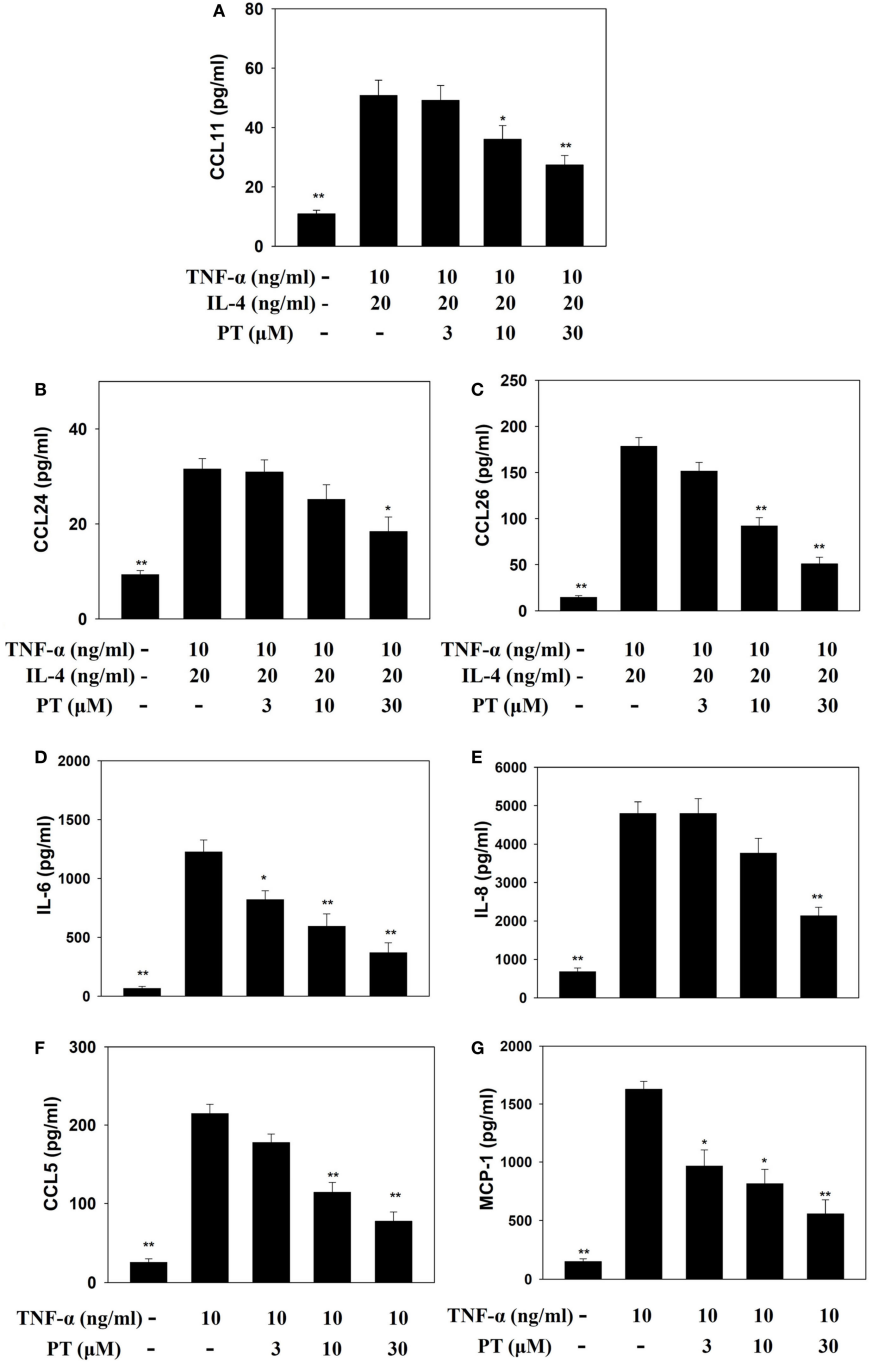
**Phloretin (PT) effects on cytokine and chemokine production in BEAS-2B cells**. Enzyme-linked immunosorbent assay results show **(A)** CCL11, **(B)** CCL24, **(C)** CCL26, **(D)** IL-6, **(E)** IL-8, **(F)** CCL5, and **(G)** MCP-1 levels in BEAS-2B cells treated with tumor necrosis factor-α (TNF-α), IL-4, and/or PT. The data represent the mean ± SEM; *p < 0.05, **p < 0.01, compared to BEAS-2B cells stimulated with TNF-α alone or TNF-α and IL-4. Three independent experiments were analyzed and compared with TNF-α alone or TNF-α and IL-4.

Phloretin could decrease IL-6, IL-8, CCL5, and MCP-1 levels in TNF-α-activated BEAS-2B cells. When BEAS-2B cells were stimulated with TNF-α and IL-4, PT also significantly inhibited CCL11, CCL24, and CCL26 production (Figure 7).

### PT Reduced Monocytic Cell Adhesion to BEAS-2B Cells

**Figure 8:**
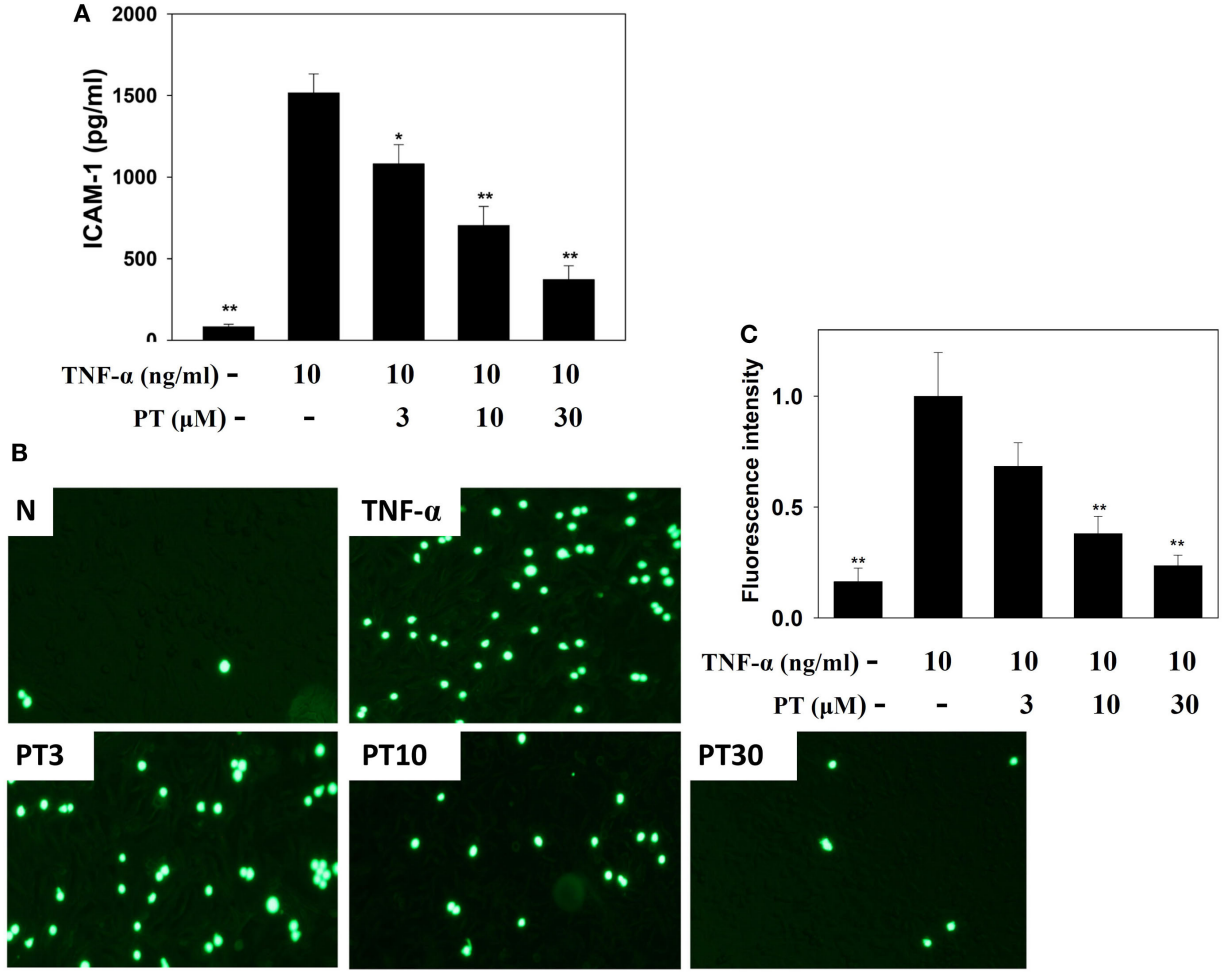
**Phloretin (PT) inhibited THP-1 cell adherence to the activated BEAS-2B cells**. **(A)** PT decreased the levels of intercellular adhesion molecule 1 (ICAM-1) in BEAS-2B cells activated with tumor necrosis factor-α (TNF-α). The data represent the mean ± SEM; *p < 0.05, **p < 0.01, compared to BEAS-2B cells stimulated with TNF-α. **(B)** Fluorescence microscopy images of THP-1 cells labeled with calcein AM and mixed with normal (N) and TNF-α-activated BEAS-2B cells, in the absence or presence of PT. **(C)** Fluorescence intensity of monocytic cell adhesion to BEAS-2B cells. The data represent the mean ± SEM; *p < 0.05, **p < 0.01, compared to BEAS-2B cells stimulated with TNF-α alone or TNF-α and IL-4. Three independent experiments were analyzed and compared with TNF-α alone or TNF-α and IL-4.

Phloretin significantly decreased ICAM-1 expression in TNF-α-activated BEAS-2B cells (Figure 8A). We also evaluated whether PT could inhibit the attachment of THP-1 monocyte cells to inflammatory BEAS-2B cells. TNF-α-stimulated BEAS-2B cells were cocultured with THP-1 cells (stained with calcein AM). PT treatment significantly reduced THP-1 cell adherence to TNF-α-activated BEAS-2B cells (Figures 8B,C).

### Effect of PT on ROS Production

**Figure 9:**
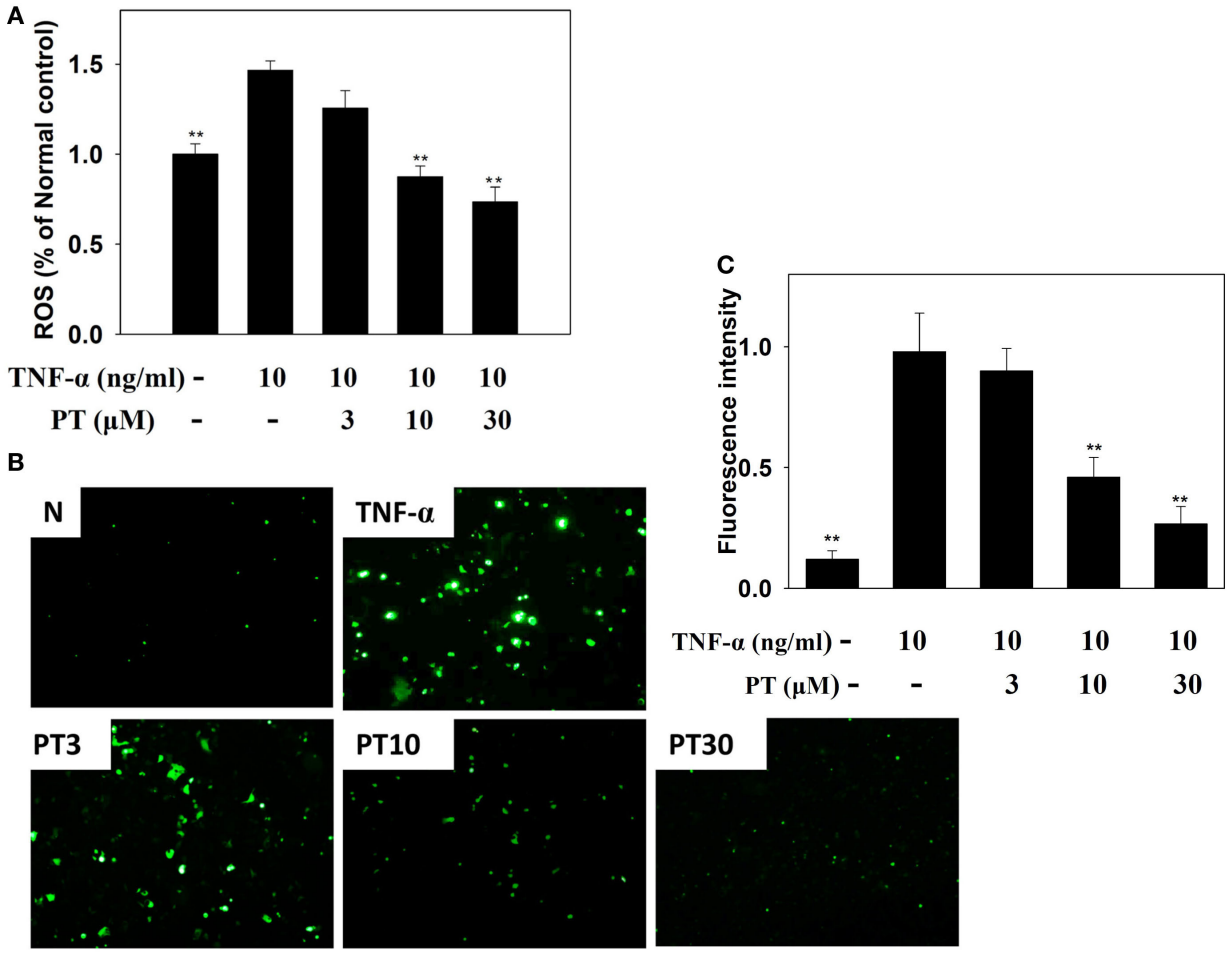
**Phloretin (PT) effects on reactive oxygen species (ROS) production in activated BEAS-2B cells**. **(A)** Percentages of ROS detected in tumor necrosis factor-α (TNF-α)-activated BEAS-2B cells in the absence or presence of PT, compared to untreated cells (N); **(B)** fluorescence microscopy images of intracellular ROS. **(C)** Fluorescence intensity of intracellular ROS. Data represent the mean ± SEM; **p < 0.01, compared to BEAS-2B cells stimulated with TNF-α alone. Three independent experiments were analyzed and compared with TNF-α alone or TNF-α and IL-4.

BEAS-2B cells were stained with DCFH-DA, the cells were lysed, and ROS production was quantified with a Multi-Mode Microplate Reader. We found that PT reduced ROS production in TNF-α-activated BEAS-2B cells (Figure 9A). Furthermore, we examined intracellular ROS in intact cells with a fluorescence microscope. We observed that PT attenuated intracellular ROS expression in TNF-α-activated BEAS-2B cells (Figures 9B,C).

## Discussion

Phloretin is a bioactive flavonoid derived from the apple tree. It has anti-inflammatory effects in inflammatory macrophages and human lung epithelial cells (4). Previous studies have shown that PT could regulate glucose transporters and increase lipolysis in 3T3-L1 adipocytes (10, 18). Our previous study found that PT improved the inflammatory and oxidative stress in LPS-induced acute lung injury mice (12). In the present study, we evaluated the hypothesis that PT might ameliorate the pathological manifestations of asthma in an asthmatic mouse model. These results suggested that PT could ameliorate local asthma symptoms in the lung by attenuating Th2 cell activity in the immune system of this experimental asthmatic model. PT acted by blocking the inflammatory response and alleviating oxidative stress.

Oxidative stress plays an important role in the development of several chronic diseases, including cardiovascular disorders, cancer, diabetes, and asthma (19). Asthma is a disorder involving pulmonary inflammation and allergic reaction; thus, excessive oxidative stress would exacerbate airway inflammation and cause aggravated bronchospasms that could worsen lung tissue damage (20). In several animal studies, excess ROS was reported to increase inflammatory cell infiltration into the lungs, stimulate extracellular matrix protein production, and stimulate proinflammatory cytokine production in airway epithelial cells (21, 22). It was previously shown that natural antioxidants could improve the pathological manifestations of asthma by blocking oxidative stress in asthmatic mice (4, 23). Other studies showed that PT had antioxidative effects on inflammatory colorectal epithelial cells and attenuated oxidative reactions in rats that underwent cecal ligation and puncture-induced sepsis (24, 25). PT also improved oxidative injury in a rat model of cerebral ischemia (24).

Malondialdehyde is a lipid peroxidation marker. It is derived from prostaglandin biosynthesis in cells during oxidative stress (20). Antioxidant enzymes, such as GSH, catalase, and superoxide dismutase, offer protection from oxidation by suppressing the chronic inflammatory response and preventing the deterioration of lung tissue in allergic asthma (20, 22). In the current study, we demonstrated that PT significantly decreased MDA and increased GSH, which ameliorated oxidative stress in lung tissues of asthmatic mice. We also found that PT could increase nuclear Nrf2 expression, which contributed to increasing HO-1 expression, and provided protection from oxidation in asthmatic lungs. Furthermore, our findings demonstrated that PT could suppress ROS production in TNF-α-activated human tracheal epithelial cells. Hence, we showed that PT could provide antioxidative effects to ameliorate lung injury in asthma.

Airway hyperresponsiveness is an important feature of asthma. Allergens might induce an acute asthma attack, which presents as bronchoconstriction and severe shortness of breath (1). Clinically, asthma is diagnosed in both children and adults by measuring the AHR, evaluating the airflow, and investigating the pathological features of asthma (22, 26). In patients with asthma, oxidative stress enhances the production of proinflammatory mediators, increases the AHR, and stimulates mucin secretion in the airways (27). PT significantly reduced elevations in inflammatory mediators in BALF and lung tissue of OVA-induced asthmatic mice and decreased proinflammatory cytokines and chemokines in human tracheal epithelial cells; thus, PT could ameliorate lung damage in asthma. Previous studies suggested that IL-13, which is a Th2-associated cytokine, could aggravate AHR in patients with asthma (28). In patients with asthma, where airway function is deteriorated and inflammatory responses are induced, high IL-13 levels have been observed in BALF and lung (29). We found that PT treatment reduced the IL-13 levels in BALF and suppressed IL-13 gene expression in the lungs of asthmatic mice, which may have contributed to AHR attenuation.

Excessive secretion of Th2 cytokines exacerbates the severity of an allergic response by increasing inflammatory cell infiltration and inducing goblet cell hyperplasia, which in turn, causes excess mucus secretion in asthmatic lungs (4, 30). In patients with asthma, Th2 cells release elevated IL-5 levels, which increase eosinophil differentiation in bone marrow cells (6). Eotaxins (CCL11 CCL24, and CCL26) are thought to attract eosinophil migration into inflamed lung tissues, and the release of more inflammatory mediators by activated eosinophils increases lung tissue injury (5, 29). Previous study demonstrated that eosinophil would secret major basic protein for induced mast cell degranulation to exacerbate allergy response (6). Eosinophil also released eosinophil cationic protein and eosinophil peroxidase to create the transmembrane channels for cytotoxic molecules into the cell (29). Hence, eosinophils accumulation in lung tissue would cause serious asthmatic allergy and inflammatory response. Eosinophils also induced the development of airway remodeling (5). Our results demonstrated that PT could inhibit IL-5, CCL11, and CCL24 expression in the lungs and BALF of asthmatic mice. PT also decreased the levels of CCL11, CCL24, and CCL26 in inflamed tracheal epithelial cells. Additionally, PT also reduced ICAM-1 in inflamed tracheal epithelial cells, which reduced their ability to adhere to inflammatory cells in lung tissues. Thus, PT could reduce eosinophil infiltration into lung tissue by blocking IL-5 and eotaxin production. Furthermore, activated macrophage also released more inflammatory cytokines to destroy the function of lung cell (31). In our murine asthma model, we found that asthma mice did not significantly increase macrophage infiltration in BALF compared to normal mice. Asthmatic mice only treated with 20 mg/kg PT had significantly reduced numbers of macrophage compared to the OVA group. However, eosinophil activation and proliferation would induce the development of asthma disease (4). Hence, macrophages did not significantly affect asthma symptoms in PT-treated asthmatic mice.

Moreover, IL-4 can activate B cells to secrete IgE and bind to mast cells; this binding activates the complex of allergic responsive IgE and mast cells, and they release leukotrienes and histamine; these factors cause acute allergic and inflammatory reactions in patients with asthma (28). Importantly, PT can decrease Th2 cell production of IL-4 to reduce the pathological characteristics of asthma.

In patients with asthma, allergens stimulate airways and induce smooth muscle cell proliferation, which causes airway narrowing and tracheal goblet cell proliferation, which aggravates mucus secretion (32). These effects cause shortness of breath and difficulty in breathing. Furthermore, IL-4 and IL-13 can activate and stimulate goblet cell hyperplasia in the trachea (29). Our observations in asthmatic mice showed that PT reduced goblet cell hyperplasia and suppressed excessive mucus secretion, which improved mucus congestion and airway asphyxia by blocking the expression of IL-4 and IL-13 in BALF and lung tissue.

Glucose transporter 2 (GLUT2) plays an important role for sensing the levels intestinal glucose (33). In adipocyte and hepatocyte, more glucose accumulate could convert to lipid. Obesity could increase the developing asthma in obese adults and children (34). PT (an inhibitor of GLUT2 transporter) could decrease glucose into intestinal serosal fluid (35). However, we did not evidence whether PT modulated glucose levels to improve asthma symptom in this experimental asthma model. Collectively, we demonstrated that PT significantly reduced eosinophil infiltration and mucus hypersecretion by suppressing eotaxin and Th2 cytokine production in asthmatic mice. These results suggested that PT has the potential to attenuate oxidative stress and inflammation in asthma.

## Ethics Statement

Animal care and experimental procedures were performed in accordance with the guidelines of the Laboratory Animal Care Committee of Chang Gung University of Science and Technology (IACUC approval number: 2014-023). Female BALB/c mice were obtained from the National Laboratory Animal Center in Taiwan. Mice were kept and maintained in air-conventional animal housing on a 12 h light/dark cycle. Before the experiment, the mice adapt the experimental environment of at least 1 week. The care and housing of experimental animals were approved in accordance with the guidelines of the Laboratory Animal Care Committee of Chang Gung University of Science.

## Author Contributions

Designed and performed the experiments: C-JL, L-WF, and W-CH; analysis and interpretation of data: L-WF and W-CH; drafting the manuscript: C-JL and W-CH.

## Conflict of Interest Statement

The authors declare that the research was conducted in the absence of any commercial or financial relationships that could be construed as a potential conflict of interest.
