# Corrigendum: Phloretin Attenuates Allergic Airway Inflammation and Oxidative Stress in Asthmatic Mice

**DOI:** 10.3389/fimmu.2020.582838

**Published:** 2020-10-19

**Authors:** Wen-Chung Huang, Li-Wen Fang, Chian-Jiun Liou

**Affiliations:** ^1^Graduate Institute of Health Industry Technology, Research Center for Industry of Human Ecology, Research Center for Chinese Herbal Medicine, College of Human Ecology, Chang Gung University of Science and Technology, Taoyuan, Taiwan; ^2^Division of Allergy, Asthma, and Rheumatology, Department of Pediatrics, Chang Gung Memorial Hospital, Taoyuan, Taiwan; ^3^Department of Nutrition, I-Shou University, Kaohsiung, Taiwan; ^4^Department of Nursing, Research Center for Chinese Herbal Medicine, Chang Gung University of Science and Technology, Taoyuan, Taiwan

**Keywords:** asthma, cytokine, eosinophil, oxidative stress, phloretin

In the original article, there was a mistake in Figure 4 as published. There was an unintentional error in the table composition of Figure 4C. The corrected Figure 4 appears below.

**Figure 4:**
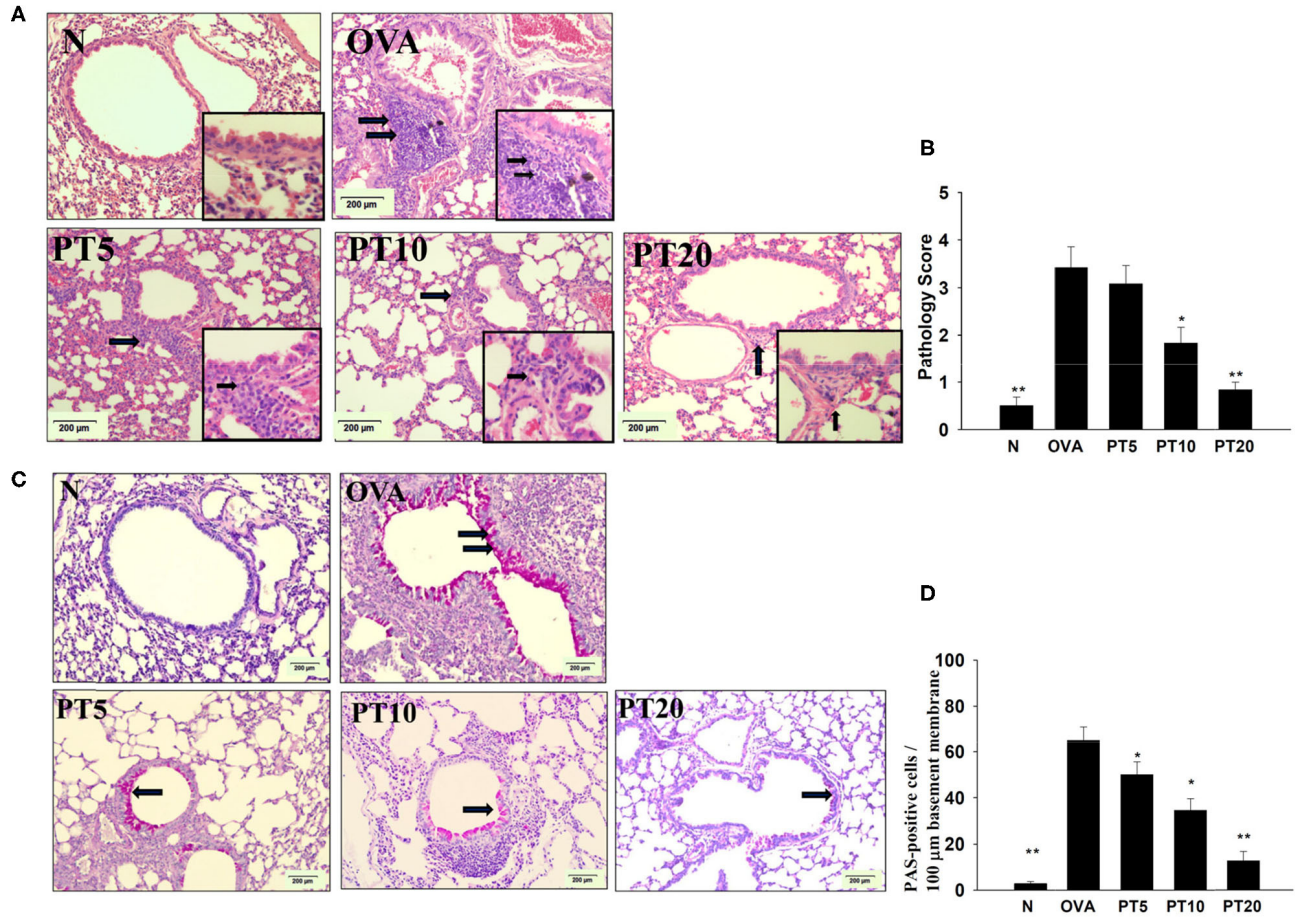
**Phloretin (PT) effects on asthmatic lung tissue**. Histological sections of lung tissues from normal (N) and OVA-stimulated (OVA) mice, without or with PT (PT5-20) treatment. **(A)** PT reduced eosinophil infiltration; eosinophils are indicated with arrows (hematoxylin and eosin stain; 200 × magnification). Amplification sections (400 × magnification) were shown for the indicated areas. **(B)** Scoring of inflammation *via* pathological evaluation of inflammatory cell infiltration in lung sections. **(C)** Periodic acid-Schiff (PAS)-stained lung sections show goblet cell hyperplasia; goblet cells are indicated with arrows (200 × magnification). **(D)** Results were expressed as the number of PAS-positive cells per 100 μm of basement membrane. All data are presented as the means ± SEM. ^*^p < 0.05 compared to the OVA control group. ^**^p < 0.01 compared to the OVA control group. Three independent experiments were analyzed and compared with the OVA-sensitive mice.

The authors apologize for this error and state that this does not change the scientific conclusions of the article in any way. The original article has been updated.

